# The interplay between the PI3K/AKT pathway and circadian clock in physiologic and cancer‐related pathologic conditions

**DOI:** 10.1111/cpr.13608

**Published:** 2024-02-09

**Authors:** Mohammad Rafi Khezri, Hsiang‐Yin Hsueh, Somayeh Mohammadipanah, Javad Khalili Fard, Morteza Ghasemnejad‐Berenji

**Affiliations:** ^1^ Reproductive Health Research Center, Clinical Research Institute Urmia University of Medical Sciences Urmia Iran; ^2^ The Ohio State University Graduate Program in Molecular, Cellular and Developmental Biology The Ohio State University Columbus Ohio USA; ^3^ Department of Pharmacology and Toxicology, Faculty of Pharmacy Tabriz University of Medical Sciences Tabriz Iran; ^4^ Department of Pharmacology and Toxicology, Faculty of Pharmacy Urmia University of Medical Sciences Urmia Iran; ^5^ Research Center for Experimental and Applied Pharmaceutical Sciences Urmia University of Medical Sciences Urmia Iran

## Abstract

The circadian clock is responsible for the regulation of different cellular processes, and its disturbance has been linked to the development of different diseases, such as cancer. The main molecular mechanism for this issue has been linked to the crosstalk between core clock regulators and intracellular pathways responsible for cell survival. The PI3K/AKT signalling pathway is one of the most known intracellular pathways in the case of cancer initiation and progression. This pathway regulates different aspects of cell survival including proliferation, apoptosis, metabolism, and response to environmental stimuli. Accumulating evidence indicates that there is a link between the PI3K/AKT pathway activity and circadian rhythm in physiologic and cancer‐related pathogenesis. Different classes of PI3Ks and AKT isoforms are involved in regulating circadian clock components in a transcriptional and functional manner. Reversely, core clock components induce a rhythmic fashion in PI3K and AKT activity in physiologic and pathogenic conditions. The aim of this review is to re‐examine the interplay between this pathway and circadian clock components in normal condition and cancer pathogenesis, which provides a better understanding of how circadian rhythms may be involved in cancer progression.

## Graphical abstract

The PI3K/AKT signalling pathway and circadian clock components affect each other in cancer pathologic circumstance resulting in excessive cellular proliferation, apoptosis inhibition, metastasis and resistance to chemotherapy.

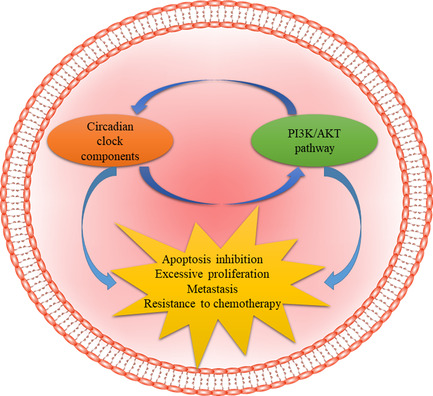

## 1 BACKGROUND

Circadian clocks are ubiquitous, cell‐autonomous timing mechanisms responsible for generating 24‐h rhythmic cycles that are conserved from unicellular organisms to humans. These internal rhythmic systems respond to metabolic and environmental stimuli to control cellular biological activities, the most important of which are sleep/wake cycles, energy metabolism, immune and hormonal activities, and cell proliferation.^1^ Alterations in circadian clocks resulting from different reasons such as sleep disturbance or chronic jet lag are closely linked to the development of a variety of pathologies including obesity, diabetes, and cancers.^2^ Particularly, genetic and environmental factors followed by circadian rhythms disruption can alter the expression and function of various oncogenes and tumour suppressors in both normal and tumour tissues to induce cancer initiation and progression.^3^, ^4^ In clinical settings, a large set of studies have been conducted to evaluate the levels of circadian clock components in patients with various cancers. The results of these studies clearly show that several circadian clock regulators are disrupted during cancer progression. However, numerous studies indicate that there is a clear association between circadian clock components and regulation of the expression of various genes, especially those involved in cell proliferation, apoptosis, and drug resistance. The phosphatidylinositol 3‐kinase (PI3K)/AKT signalling pathway, which participates as a downstream effector of G protein‐coupled receptors (GPCR) or receptor‐tyrosine kinases (RTK), is known as a crucial regulator of cell survival and its over‐activation has been detected in different cancer types.^5^ Aberrant activity of the PI3K/AKT signalling pathway affects the function of downstream targets that are involved in cell growth and proliferation. However, it seems that there is a significant and bilateral link between the PI3K/AKT signalling pathway and core clock components in different transcriptional and functional stages. Growing evidence indicate that the activity of the PI3K/AKT pathway in a variety of body organs is regulated in a rhythmic fashion. It seems that the expression and activation of AKT follows a rhythmicity in response to day‐night cycles. It is clearly understood that various isoforms of PI3K regulate expression and activity of core clock components, especially BMAL1. Indeed, AKT phosphorylates BMAL1 and CLOCK proteins at different sites, which leads to regulating their ubiquitination and translocation into the nucleus. Disrupted activity of the PI3K/AKT and core clock components may influence each other leading to progress pathologic factors involved in cancer progression, including altered apoptosis, angiogenesis, and drug resistance. This association may present a new insight into how circadian rhythm disruptions may lead to cancer initiation and progression by mediating of the PI3K/AKT pathway. This paper aims to provide an overview on the association of the PI3K/AKT pathway and circadian clock in the physiological and pathological conditions such as cancer progression in order to introduce a possible therapeutic target for cancer therapy.

## 2 THE PI3K/AKT PATHWAY

The PI3K/AKT signalling pathway accounts for a pivotal intracellular pathway involved in the progression of a wide variety of diseases, the most important of which are cancer. There is a significant correlation between the activity of the PI3K/AKT signalling pathway and the progression of different cancers including gastric, breast, ovarian, prostate, colorectal, glioblastoma, and endometrial cancers.^6^ The main effector of this pathway, PI3K, constitutes a central link between upstream stimuli and downstream signalling pathways resulting in regulation of a wide variety of cellular processes including protein synthesis, metabolism, cell survival, inflammation, motility, and angiogenesis.^6^ PI3Ks are a large family of enzymes with an ability to phosphorylate the 3′‐OH group of the inositol head group of phosphatidylinositol, which is localized on the cell membrane. There are three classes of PI3Ks in mammalian cells, namely class I, class II, and class III. Class IA PI3Ks have two main subunits including a regulatory subunit and a catalytic subunit. The catalytic subunits p110β, p110α, or p110δ are encoded by the *PIK3CB*, *PIK3CA*, and *PIK3CD* genes, respectively. Meanwhile, the regulatory subunits involve p85β, p85α, and p55γ, which are encoded by the *PIK3R2*, *PIK3R1*, and *PIK3R3* genes, respectively. Contrary to class IA, class IB PI3Ks have only one catalytic subunit, P110γ, along with two regulatory subunits, p101 and p84.^7^ Three class II PI3Ks isoforms (PI3K‐C2α, PI3K‐C2β, and PI3K‐C2γ) have crucial and non‐overlapping cellular functions. PI3K‐C2α plays a crucial role in clathrin‐mediated endocytosis, mitosis, and vesicular trafficking. PI3K‐C2β is stimulated by growth factors and is involved in mTOR signalling repression and cell migration. PI3K‐C2γ controls Akt2 activation and glycogen storage in the liver.^8^ The only member of class III PI3Ks is Vps34, which is a primordial PI3K isoform conserved from yeast to human. Vps34 associates with specific intracellular protein complexes and regulates endosomal sorting, autophagy, phagocytosis and micropinocytosis.^9^, ^10^

After being triggered by various stimuli, both GPCRs and RTKs activate PI3Ks, leading to the activation of the protein‐serine/threonine kinase AKT.^11^ It is necessary to recruit phosphatidylinositol 4,5‐bisphosphate (PIP_2_) or phosphatidylinositol 3,4,5‐trisphosphate (PIP_3_) to the plasma membrane to activate AKT, where its activation by phosphorylation at Thr‐308 or Ser‐473 sites occurs.^12^ To date, three AKT isoforms have been identified in mammalian cells: AKT1, AKT2 and AKT3.^12^ AKT1 and AKT2 are commonly expressed in different human tissues. However, AKT2 is highly expressed in insulin‐responsive tissues such as the muscle, liver, and pancreas. Meanwhile, AKT3 isoform exhibits a more restricted tissue distribution with a high expression in the brain.^13^ Contrary to in vitro studies, emerging evidence from in vivo studies indicate an isoform‐specific biological function for different AKT isoforms.^14^ In fibroblasts, AKT2 has been shown to be activated at both early endosomes and plasma membrane, whereas the activity of AKT1 and AKT3 was restricted to the cell membrane. In addition, it was shown that AKT isoforms selectivity to binding to 3‐phosphoinositide depends on the short linker region between the PH and catalytic domains within their structure. AKT1 and AKT3 isoforms are able to bind to PIP_3_, whereas AKT2 shows a strong selectivity for both PIP_2_ and PIP_3_.^15^

As for the activation mechanism of the PI3K/AKT pathway, more than 276 AKT substrates and downstream targets have been identified to date (www.kinasenet.ca), including apoptosis inducers (BAD), cell cycle progression inhibitors (p21 and p27), GAPs (TSC2 and AS160), protein kinases (glycogen synthase kinase 3β (GSK3β)) and a group of the forkhead box protein O (FOXO) transcription factors. Hyperactivity of the PI3K/AKT signalling pathway due to gene mutations in PI3K or AKT isoforms as well as their regulators has been detected in different types of cancer. The PIK3CA gene highly amplified in gastric, prostate, thyroid, ovarian, cervical, and several other cancers.^16^ Copy numbers of the PIK3CB gene are gained frequently in thyroid, lung, and lymphoma. Indeed, the amplification of PIK3CD gene is more enriched in glioblastoma.^17^, ^18^ Clinically, elevated expression of PIK3CA has been shown to be correlated with tumour invasion and poor prognosis among cancer patients. Moreover, overexpression of PIK3CA gene has been associated with lymph node metastasis in different cancers, including colorectal and gastric cancers.^19^, ^20^, ^21^ Within the structure of PI3Kα, the majority of mutations are detected at residues lying at the interfaces between p110α and p85α or other domains within the catalytic subunit, which is known as one of the most common mutations in different types of cancers, especially breast cancer.^22^, ^23^ High copy of AKT1 gene has been detected in several cancers including breast and lung cancers, which has been linked to increased resistance to cisplatin.^24^, ^25^ Amplification in the AKT2 gene has been detected in ovarian carcinomas, hepatocellular, gastric, colorectal, and breast cancers.^26^, ^27^, ^28^ Additionally, a hot‐spot Akt1‐E17K mutation has been detected in pleckstrin homology domain of AKT1 which was linked to progression of ovarian and breast cancers.^29^, ^30^ This is the most common mutation associated with AKT1, occurs in AKT2 and AKT3 at a much lower frequency in human cancers. Notably, gene mutations in the Akt3‐E17K has been linked to melanoma initiation as well as other developmental disorders such as asymmetric cortical dysplasia.^31^, ^32^

## 3 MECHANISMS OF THE PI3K/AKT PATHWAY ACTIVATION

As described, stimulation of RTKs and GPCRs mainly contributes to activation of class IA and IB PI3K isoforms, respectively.^33^ The main function of PI3Ks is to catalyse the phosphorylation of phosphatidylinositol at the 3′ position of the inositol ring resulting in the generation of D3 phosphorylated phosphoinositides, mainly PIP_2_ and PIP_3_.^34^ There are a couple of specific target proteins for PIP_2_ and PIP_3_, which feature specific binding domains leading to their translocation to the cell membrane followed by their activation.^34^ Accordingly, PIP_2_ and PIP_3_, as the phosphoinositide products of PI3K, bind to the PH domain of AKT resulting in translocation and localization of AKT to the cell membrane. However, there are two types of AKT activation. Full activation of AKT needs its phosphorylation at two sites including the activation loop (T308 for AKT1), and the C‐terminus region (S473 for AKT1). Partial activation of AKT occurs when AKT is phosphorylated at T308 by 3′‐phosphoinositide‐dependent kinase‐1 (PDK‐1), whose activity is also regulated by PI3K with the production of PIP_3_.^35^

In addition to PI3K‐mediated AKT activation, there are several other mechanisms in a PI3K‐independent manner. Changes in intracellular levels of calcium as well as protein kinase A (PKA) and β‐adrenergic receptors agonists have all been shown to induce AKT activity that was not altered by PI3K inhibitors.^36^, ^37^ Indeed, mTOR complex 2 (mTORC2) is involved in the activity of AKT via phosphorylation at S473.^38^ Also, mTORC2 phosphorylates AKT at T450 which regulates AKT folding and maturation.^39^ In addition to mentioned routes, AKT activation may occur in response to a variety of cellular stresses, including administration of ultraviolet light, heat shock, hypoxia, ischemia, hypoglycemia, and oxidative stress (reviewed at^40^).

## 4 CIRCADIAN RHYTHM PROTEINS

### 4.1 A brief look at circadian rhythm

At the beginning of the 18th century, French scientist, Mairan, found that light did not affect the fluctuation activity of mimosa leaf, indicating that there may be an endogenous rhythm that regulate the metabolic activities of plants. The term circadian rhythm was first used by Halberg to reveal the near‐24‐hour (h) endogenous rhythmicity of biological processes within the organisms in response to the earth's daily rotation cycle.^41^ Particularly, the daily light–dark cycle acts as a primary external synchronizer for organisms' circadian rhythm. In mammals, light processing takes place within the eye. This information is then transmitted via the retinohypothalamic tract to the suprachiasmatic nucleus (SCN) in the hypothalamus, which serves as the primary internal pacemaker.^42^ The SCN bilateral nuclei contains approximately 10,000 neurons that display as a cell‐autonomous circadian oscillator. In addition to the master clock in SCN, several other functional nuclei have been known to act as semiautonomous clocks as slave oscillators (including bed nucleus of the stria terminalis, paraventricular nucleus, amygdala, preoptic area, nucleus accumbens) or as semiautonomous oscillation clocks (including dorsomedial hypothalamus, olfactory bulb, arcuate nucleus), both synchronized by the SCN.^43^ The central pacemaker clock in the brain coordinates different peripheral clocks expressed in almost every human organ, such as liver, lungs, heart, and skeletal muscle. To better understand the gene expression in response to day‐night cycle, the gene expression across 64 distinct tissues and brain regions of male baboons was examined at two‐hour intervals throughout a 24‐h day. Interestingly, the data revealed that rhythmic transcripts exist in all tissues, but the number of cycling transcripts varies from approximately 200 to over 3000 in a given tissue. 96.6% out of the 11,000 genes that are commonly expressed in all tissues showed 24‐h rhythmicity in at least one tissue.^44^ The coordination of the peripheral rhythmic clocks is governed not only in a vertical and hierarchical manner through the suprachiasmatic nucleus (SCN) by means of the peripheral nervous system (PNS), but it also undergoes horizontal control through both humoral and non‐humoral routes. Particularly, SCN regulates peripheral oscillators activity via different mechanisms, including autonomic innervation of peripheral tissues, body temperature, endocrine signalling (glucocorticoids), and feeding‐related cues. Both sympathetic and parasympathetic pathways are necessary for the neural synchronization of peripheral oscillators.^45^ Delivery of the entraining signal for the submandibular salivary glands from SCN projections occurs via the paraventricular nucleus–superior cervical ganglia (PVN‐SCG) pathway.^46^ The information to oscillators in the liver and adrenal gland is provided from SCN‐derived autonomic pathways.^47^ Furthermore, the sympathetic system modulates the adrenal sensitivity to adrenocorticotropic hormone and the release of glucocorticoids.^47^, ^48^ In the cortex and medulla regions of the adrenal gland, cellular oscillators respond to SCN‐derived neural inputs.^49^

### 4.2 Regulators of circadian rhythm

**FIGURE 1:**
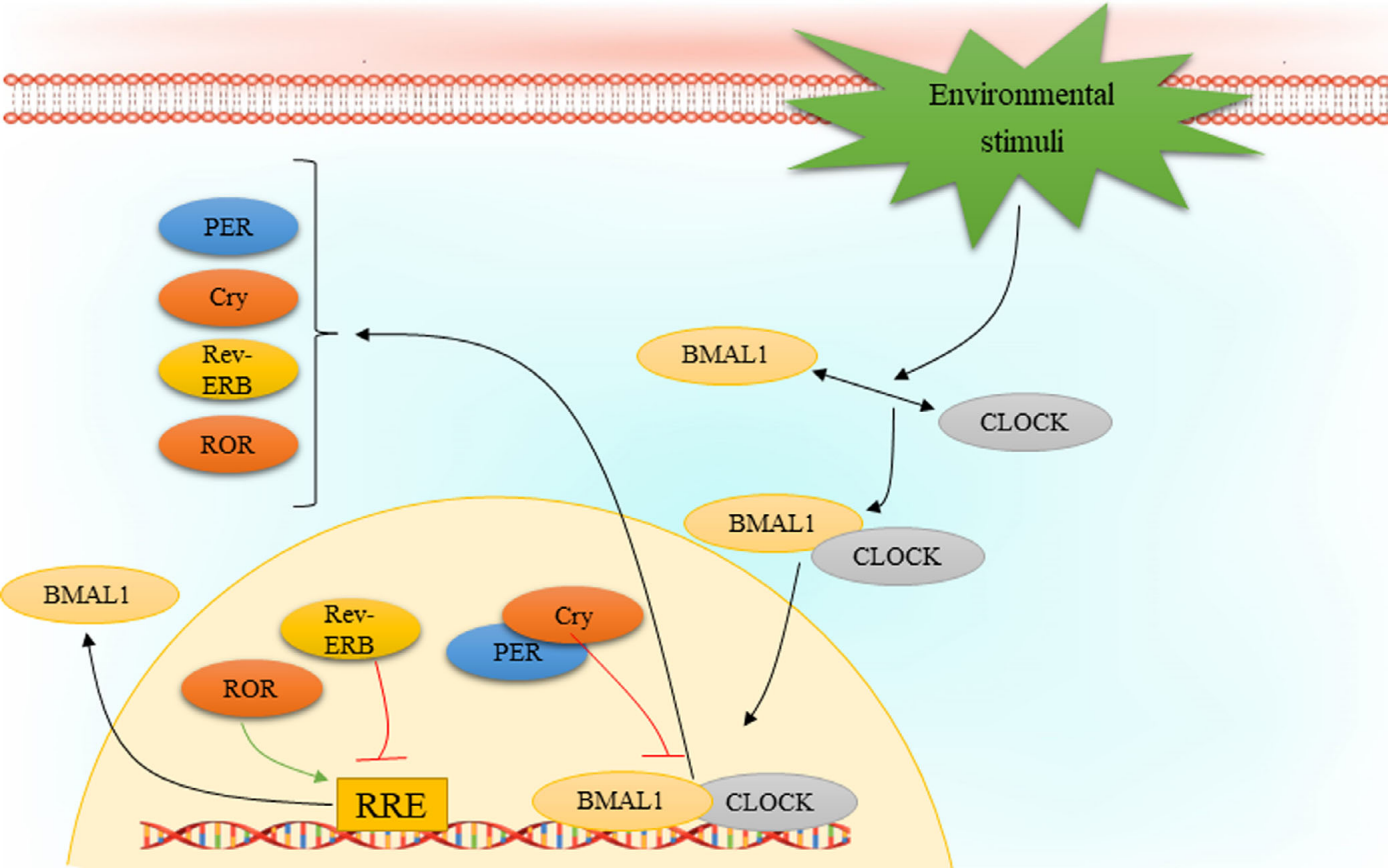
Circadian clock autoregulation mechanism. Different environmental stimuli, such as light, contribute to BMAL1 and CLOCK heterodimerization. On the one hand, BMAL1:CLOCK complex enters the nucleus and its binding to E‐box regions of specific genes occurs followed by transcription of other core clock regulators, PER, Cry, Rev‐ERB and ROR. Accumulation of Cry and PER in the nucleus results in inhibition of the transcriptional activity of CLOCK:BMAL1 complex. On the other hand, ROR and Rev‐ERB regulate the expression of BMAL1. BMAL1, brain and muscle Arnt‐like protein 1; CLOCK, circadian locomotor output cycles kaput; Cry, cryptochrome; PER, period; ROR, retinoic acid‐related orphan receptor.

The circadian rhythm is regulated by a wide variety of transcription factors leading to auto‐regulatory transcription‐translation feedback loops (TTFLs). The most important players in regulation of TTFL are circadian locomotor output cycles kaput (CLOCK) and brain and muscle Arnt‐like protein 1 (BMAL1, also known as ARNTL). Particularly, transcription of CLOCK and BMAL1 genes results in the heterodimerization and formation of the BMAL1: CLOCK complex, which is translocated from cytoplasm into the nucleus and binds to canonical Enhancer Box (E‐Box)‐sequences that consists of the consensus sequence CACGTG or non‐canonical E‐Boxes of genes regulated by clock. The BMAL1: CLOCK complex has a set of target genes, among them it promotes the expression of several factors that form the negative regulators of molecular clock, such as cryptochrome (CRY1, CRY2) and period (PER1, PER2, PER3). Formation of a complex containing CRY and PER in the cytoplasm and its translocation and accumulation in the nucleus contributes to inhibit the activity of BMAL1: CLOCK complex. However, there is a multi‐protein E3 ubiquitin ligase complex that regulates PER and CRY stability. Three main components of this complex are Skp1, Cullin, and F‐box protein that involve β‐Transducin Repeat Containing E3 Ubiquitin Protein Ligase (β‐TrCP) and F‐Box and Leucine Rich Repeat Protein 3 (FBXL3), respectively. The adenosine 3′,5′‐monophosphate (AMP) kinase (AMPK) and casein kinase 1ε/δ (CK1ε/δ) phosphorylate the proteins CRY1 and PER at S661, S663 and S714, respectively The phosphorylation event triggers their targeted poly‐ubiquitination through specific ubiquitin ligase complexes. Consequently, the CRY and PER proteins undergo degradation facilitated by the 26 S proteasome complex. A decrement in CRY and PER protein levels relieves the inhibition of BMAL1: CLOCK activity, thus permitting to start a new oscillation. Moreover, there are several other key regulators of the circadian clock, including the nuclear receptors REV‐ERBα and REV‐ERBβ, as well as the retinoic acid orphan receptor (ROR) (RORα, RORβ, and RORγ) that form another feedback loop. REV‐ERBs act as transcriptional inhibitor of BMAL1 expression, meanwhile, RORs regulate the BMAL1 expression positively through binding to sites Retinoic acid receptor‐related Orphan Receptor Element (RORE) elements that exist in the BMAL1 gene promoter.^43^ In addition to transcriptional regulation of circadian clock, its regulation might also encompass various other mechanisms, including intracellular calcium flux, membrane depolarization, and activation of cyclic‐AMP (cAMP) signalling. Studies have shown that the buildup of intracellular calcium plays a crucial role in generating neuronal firing rhythms within the SCN.^50^ Furthermore, the rhythmic expression of circadian clock regulators in SCN neurons requires processes such as membrane depolarization, periodic calcium influx, and daily activation of cAMP.^51^, ^52^ Indeed, the phosphorylation followed by activation of the calcium/cAMP response element binding protein (CREB) can mediate such effects via binding to calcium/cAMP regulatory elements (CREs) on DNA. It has been reported that CRE sequences can be found in the gene promoters of several clock components, such as PER1 and PER2.^52^, ^53^ Figure 1 depicts the auto‐regulation process of circadian rhythm.

### 4.3 Circadian clock in cancer

Increasing evidence indicate the potential association between mammalian circadian rhythm and cancer pathogenesis. Nowadays, approximately 80% of the world's population is exposed to artificial light during the night, and 18–20% of USA and Europe workers are involved in rotating shift work, which makes them vulnerable to a set of rhythm pathologies, including cancer.^54^ There are several epidemiological studies suggesting the association between night shift work or chronic jet lag and increased incidence and development of different cancer types especially common ones, such as breast, prostate, colorectal, lung, and skin cancers.^2^ As of today, genetic studies have found a significant alteration in the expression of circadian clock genes in different types of cancer (summarized in Table 1). In a molecular inspection, it has become clear during the past decade that circadian clock components affect cell growth, which can be a reason for cancer initiation and progression.^55^ Altered expression of circadian clock components can influence cell cycle, DNA repair, autophagy, apoptosis, and other oncogenic pathways resulting in excessive proliferation, apoptosis inhibition, metastasis, enhanced angiogenesis, immune evasion, and resistance to chemotherapy hallmarks of cancer.^56^ Genetic inactivation of *Clock* and/or *Bmal1* has been found to induce proliferative and malignant phenotypes in various cancers, such as colon cancer,^57^ tongue squamous cell carcinoma,^58^ pancreatic cancer,^59^ lung adenocarcinoma,^4^ breast cancer,^60^ hepatocellular carcinoma,^61^ nasopharyngeal carcinoma (NPC),^62^ and glioblastoma (GBM).^63^ Study has shown that overexpression of mentioned circadian clock components inhibits tumour proliferation or growth rates mainly via cell cycle arrest and p53‐mediated apoptosis.^64^ Interestingly, several research indicates that the core clock genes exhibit tumour promoting activity, depending on the cancer cell type or status. For instance, BMAL1 knockdown was shown to result in a significant increase in cellular apoptosis with mitotic abnormalities in malignant pleural mesothelioma cells.^65^ Similarly, silencing of either CLOCK or BMAL1 has been shown to cause cell cycle arrest and apoptosis in murine leukaemia stem cells in acute myeloid leukaemia.^66^ In addition, BMAL1 overexpression has been linked to breast cancer cell metastasis through increasing the expression of matrix metalloproteinase 9 (MMP9).^67^

**TABLE 1:** Dysregulation of core clock components in different cancers.

Cancer type	Core clock component	Status	Main outcome	Reference
Pituitary adenoma	PER2	Upregulated	Modulates the development of pituitary adenoma	[92]
Pancreatic cancer	BMAL1	Downregulated	A prognostic indicator for survival outcomes	[93]
Pancreatic cancer	PER1, PER2, PER3, Cry2, CLOCK, and BMAL	Downregulated	Correlated with reduced survival in cancer patients	[94]
Pancreatic ductal adenocarcinoma	BMAL1	Downregulated	Correlated with tumour progression and poor prognosis	[95]
Endometrial cancer	PER3	Downregulated	–	[96]
	CLOCK	Downregulated		
Cutaneous melanoma	BMAL1, RORA, RORC, PER1, PER2, PER3, CRY2	Downregulated	Correlated with the immune cells' infiltration level, correlated patients prognosis and survival	[97]
Colon adenocarcinoma	CLOCK and CRY1	Upregulated	Correlated with prognosis and immune cell infiltration	[98]
	BMAL1, CRY2, PER1, PER3, and RORA	Downregulated		
Colorectal cancer samples	REV1 and CRY2	Upregulated	Associated with tumour immunity and prognosis	[99]
Colorectal cancer tissues	PER2, Bmal1, and Clock.	No differences were observed	–	[100]
	PER1 and PER3	Downregulated		
Colorectal cancer	Cry1	Upregulated	Correlated with tumour progression and poor prognosis	[101]
Colorectal cancer	PER1	Downregulated	Correlated with high‐grade tumours	[102]
Colorectal liver metastases	CLOCK, BMAL1, PER1, PER2, PER3, Cry1, and Cry2	Downregulated	–	[103]
Gastric cancer	PER2 and Cry1	Downregulated	Correlated with more advanced stages	[104]
Head and neck squamous cell carcinoma	PER1, PER2, PER3, CRY1, CRY2, CKIε, and BMAL1	Downregulated	Downregulated PER3, CRY2, and BMAL1 expression was correlated with more advanced cancer stages, downregulated PER3 was correlated with larger tumour size and deeper tumour invasion	[105]
Oesophageal cancer	CLOCK, PER1, PER2, PER3, CRY1, CRY2, REV‐ERBα, and RORα	Downregulated	–	[106]
Metastatic melanoma	BMAL1	Downregulated	Positively correlated with antitumor immunity and patient survival	[107]
Breast cancer	BMAL1	Upregulated	–	[108]
	CLOCK	Not changed		
Neuroblastoma	RORα and BMAL1	Downregulated	Associated with a favourable clinical outcome and stage	[109]
	REV‐ERBα	Upregulated	Correlated with a poor outcome and stage	

Collectively, it is clearly understood that core clock components are involved in regulation of the expression of a variety of intracellular and extracellular factors. Disrupted activity or expression of circadian clock factors may lead to altered cellular response to environmental stimuli resulting in initiation and progression of cancer. This data has become a reason to design new studies to introduce different therapeutic options to modulate core clock components activity to suppress cancer progression (reviewed at^68^).

## 5 THE PI3K/AKT PATHWAY AND CIRCADIAN RHYTHM COMPONENTS: PHYSIOLOGIC CONDITION

It is clearly understood that the transcription of many cellular genes are regulated by circadian clock. Under normal physiological circumstances, numerous studies have observed a rhythmic pattern in the activity of AKT across various organs. This rhythm contributes to the regulation of diverse cellular functions. One study revealed that the phosphorylation of AKT at the activation site T308 exhibits circadian fluctuations, whereas the overall levels of AKT protein remain unaffected by the circadian clock.^69^ Overexpression of BMAL1 and CLOCK together influences the levels of p‐AKT, but this effect cannot be seen by BMAL1 or CLOCK alone.^69^ These observations may reveal that AKT expression is not a direct target for BMAL1‐CLOCK complex, but several other AKT‐activating kinases can be affected by this complex leading to increased AKT phosphorylation. Interestingly, the level of one of the main kinases involved in AKT phosphorylation at T308, PDK1,^70^ has been shown to be regulated by core clock components.^71^ Circadian rhythm‐based control of metabolic states in the liver and various tissues appears to involve the PI3K/AKT signalling pathway. Notably, the removal of E4BP4, a gene regulated by the circadian clock, has demonstrated the ability to modify insulin sensitivity within the liver and muscles, primarily by inducing alterations in AKT phosphorylation. Indeed, it has been indicated that in livers of Per1,2^−/−^ mice, phospho‐AKT levels follow a ultradian (about 16 h) oscillation. Conversely, it was observed that AKT phosphorylation is not required for circadian oscillation.^72^ Interestingly, another report reveals that *Bmal1* deletion in the heart affects the metabolic responses in the liver, in a way that *Bmal1* knockdown leads to decreased insulin‐induced AKT phosphorylation in the liver leading to increased insulin resistance in the liver.^73^ On the contrary, cardiomyocyte‐specific *Bmal1* knockout has been shown to affect multiple insulin signalling components in the heart, including AKT and p85α, leading to attenuated insulin‐mediated glucose utilization.^74^ A recent study showed that insulin‐mediated lipogenesis in the liver depends on the association between BMAL1 and the PI3K/AKT signalling pathway. The results of this study revealed that liver‐specific knockdown of Bmal1 results in altered expression of lipogenic enzymes, FASN and ACC1, and these effects may be linked to AKT activity. Although in this study the direct link between AKT expression and BMAL1 was not examined, a significant link between AKT phosphorylation at S473 and Bmal1 deletion was observed.^75^ AKT is phosphorylated at T308 by phosphoinositide‐dependent kinase‐1 (PDK1). PDK1 is activated by phosphatidylinositol 3,4,5‐trisphosphate (PIP3), which is produced by PI3Ks. AKT is phosphorylated at S473 by the mammalian target of rapamycin complex 2 (mTORC2). mTORC2 is activated by PIP3 and is responsible for the phosphorylation of AKT at S473. AKT can also be phosphorylated at a third site, threonine 450 (T450), which is referred to as the turn phosphorylation site. The phosphorylation of AKT at S473 is mainly induced by mTOR complex 2 (mTORC2), in which supports the claim that BMAL1 may just regulate the levels of AKT phosphorylators but not its total levels. While phosphorylation of AKT at T308 and S473 are important sites for increasing the phosphotransferase activity of AKT, there are other sites that play a stimulatory role, including T72, S124, S129, Y176, S246, Y326, T450 and Y474, as well as potential inhibitory roles, including T34 and Y315 Thus, there are numerous sites of action and underlying mechanisms that can finely tune the enzymatic activities of AKT isoforms during circadian rhythms. It is also known that the substrate specificity of AKT may be influenced by the differential phosphorylation of the kinase.^76^

The association between circadian components and the PI3K/AKT pathway is not limited to insulin regulation. This interrelation plays a fundamental role in maintaining the well‐being of various hormones and their respective organs. A recent study has demonstrated that *Bmal1* knockout female mice exhibit impaired luteal hormone synthesis via affecting the expression of steroidogenesis‐associated genes (star, Hsd3β2, cyp19a1 in GCs, Lhcgr, star, Hsd3β2, cyp17a1) in isolated *Bmal1* interference theca and granulosa cells. However, treatment of these cells with LY294002, a relatively specific inhibitor of PI3K, led to partially rescued mRNA expressions of luteinizing hormone/choriogonadotropin receptor (Lhcgr) and Hsd3β2, along with significantly increased androstenedione and T synthesis.^77^ However, in another study, it has been reported that the expression of BMAL1 and Per2 exhibit rhythmic change in granulosa cells which were enhanced by dexamethasone synchronization. It was observed that *Bmal1* siRNA treatment of granulosa cells was accompanied with reduced levels of follicle stimulating hormone receptor (Fshr), Lhcgr, oestrogen receptor 2 (Esr2), and inhibited synthesis of progesterone and estradiol. Finally, these observations were linked to suppressed PI3K/AKT activity as *Bmal1* interference inactivated this pathway in porcine granulosa cells.^78^

**FIGURE 2:**
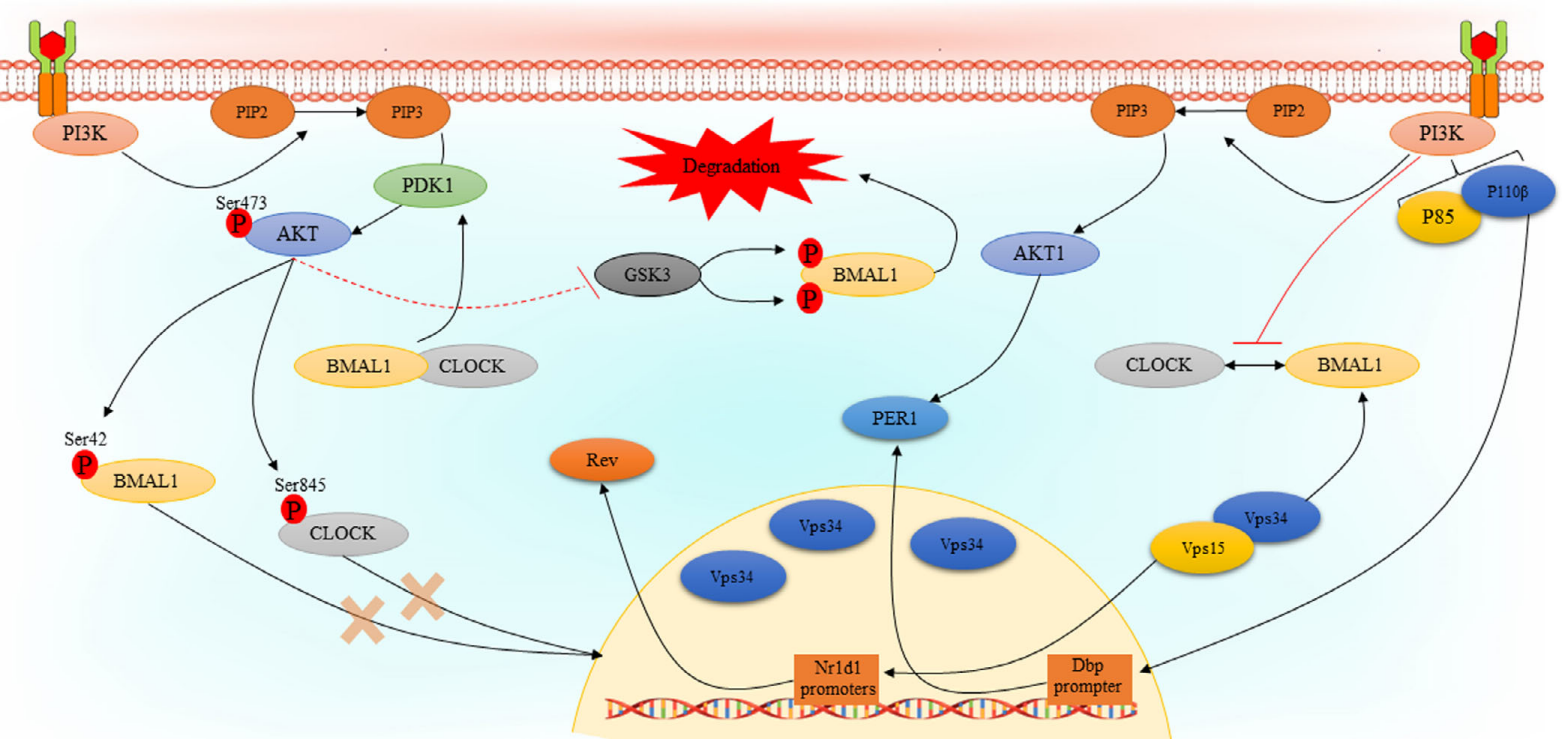
The interplay between the PI3K/AKT pathway and circadian rhythm regulators. There is a bilateral association between circadian rhythm and the PI3K/AKT signalling pathway. Phosphorylation of BMAL1 and CLOCK by AKT inhibits their entry into the nucleus. GSK3 inhibition by AKT leads to indirect reduction of BMAL1 degradation. BMAL1:CLOCK complex induces AKT activity by mediating activation of PDK1. BMAL1, brain and muscle Arnt‐like protein 1; CLOCK, circadian locomotor output cycles kaput; PI3K, phosphatidylinositol‐3‐kinase.

There are several studies that indicate the PI3K/AKT signalling pathway regulates that expression or activity of core clock components. It has been reported that Vps34, the only member of class III PI3Ks, regulates liver‐specific circadian clock to enhance rhythmic de novo purine synthesis. Vps15‐mutant mice with depleted Vps34 and subsequent autophagy block exhibited a significant change in transcript levels of Nr1d1 gene which encodes Rev‐Erbα. Interestingly, as Vps34 plays a central role in autophagy and endocytosis, it was hypothesized that prolonged dysfunction of Vps34 may influence the expression and/or turnover of BMAL1 protein. Furthermore, treatment of mouse embryonic fibroblasts (MEF) with specific Vps34 inhibitors did not affect Rev‐Erbα expression, indicating kinase‐independent role of Vps34 in clock control. In addition, it was observed that Vps34 does not affect BMAL1 activity. Also, it was found that Vps15, as Vps34 regulatory protein kinase, interacts with and co‐activates BMAL1 independently of Vps34.^79^ In another study, a different mechanism has been introduced in the case of how the PI3K regulates circadian rhythms by mediating of core clock genes. In this study, it was shown that treatment of NIH 3 T3 cells with LY294002 results in reduced the promoter activity and mRNA levels of Dbp, which is a BMAL1: CLOCK target gene and activates Per1 gene transcription by binding to its promoter. In a closer inspection, this effect was linked to p110β subunit of the PI3K, as it was observed that knockdown of p110β, but not p110α, significantly suppressed the expression levels of Dbp mRNA. Further analyses revealed that PI3K‐dependent Dbp expression may be mediated by its effect on BMAL1: CLOCK recruitment to the E‐box regions. It was observed that inhibition of the PI3K does not affect the levels of neither BMAL1 nor CLOCK, however, its effect may be mediated by inhibition of their heterodimerization.^80^ There are a couple of studies that indicate regulation of the activities of core clock components may be regulated by AKT isoforms. In a study by Luciano and co‐workers, the effects of *Akt1* knockdown on the expression of core clock components in heart, liver, orate, and mouse lung endothelial cells (MLECs) were examined. In the heart tissues of *Akt1* ^*−/−*^ mice, there was a significant change in REVα expression, but not other circadian clock regulators. As AKT2 is the main AKT isoform in the liver, *Akt1* knockdown did not affect the expression of core clock components in the liver. *Akt1* knockdown was accompanied with increased PER2, REVα, and Dbp, decreased BMAL1 and loss of CLOCK rhythmicity in aorta.^81^ Mechanistically, phosphorylation of CLOCK may be a central way for AKT‐mediated regulation of circadian rhythms.^82^ It has been clearly understood that AKT phosphorylates CLOCK at S845 within the RXRXXpS motif, which is conserved among mammals and regulates intricate aspects of circadian rhythms. Interestingly, it has been reported that CLOCK S845 phosphorylation does not affect its stability but controls CLOCK translocation into the nucleus via its interaction with 14–3‐3 proteins. Indeed, CLOCK S845A mice did not exhibit a behavioural defect but showed an alteration in circadian gene expression in the heart, skeletal muscle, and liver.^83^ In addition to CLOCK, the same observations have been found in the case of BMAL1, indicating that BMAL1 phosphorylation at S42 by AKT inhibits its accumulation in the nucleus via binding to 14–3‐3 proteins.^84^ In contrast to these results, another study showed that activation of AKT leads to a substantial increase in the expression of BMAL1, while having no such impact on CLOCK in HEK 293 cells, this effect is likely achieved through a reduction in the degradation of BMAL1. Further analyses indicated that regulation of glucose synthase kinase‐3 (GSK3) activity by AKT leads to BMAL1 phosphorylation at S17 and T21 sites resulting in its priming for ubiquitylation and degradation.^85^ However, the main issue in this study was the lack of information on whether activation or inactivation of GSK3 by AKT leads to BMAL1 ubiquitinylation and instability. Figure 2 represents the association between the PI3K/AKT pathway and circadian clock components.

In general, the results from existing studies clearly show that there is a bilateral regulatory relationship between the PI3K/AKT pathway and activity of core clock genes during circadian oscillation. Table 2 summarizes the interplay between the PI3K/AKT pathway and circadian clock components in physiologic condition.

**TABLE 2:** Crosstalk between the PI3K/AKT pathway and core clock components in physiologic and cancer‐related pathologic conditions.

Condition	Model	Result	Main outcome	Reference
Physiologic	Adult guinea pig cardiomyocyte	Over‐expressions of CLOCK‐BMAL1 induces AKT phosphorylation	Regulate the expression of L‐type calcium channels	[69]
	*Bmal1*‐knockout mice	Inhibited AKT and nitric oxide signalling	*Bmal1*‐knockout exhibit endothelial dysfunction	[110]
	E4BP4 transgenic mice	Reduced AKT phosphorylation and activation	Inhibited insulin pathway and elevated free fatty acid flux from the liver	[71]
	Per1,2^−/−^ mice	Observed ultradian oscillations of phospho‐AKT levels	–	[72]
	Heart‐specific *Bmal1*‐deficient mice	Altered AKT phosphorylation in the liver	Induced hepatic insulin resistance	[73]
	Cardiomyocyte‐specific *Bmal1* knockout	Inhibited AKT and p85α activity	Attenuated insulin‐mediated glucose utilization	[74]
	*Bmal1* ^−/−^ and acute liver‐specific *Bmal1*‐depleted mice	Impaired mTORC2/AKT activity	Decreased levels of de novo lipogenesis and lipogenic enzymes	[75]
	*Bmal1* interference GCs and TCs and *Bmal1* knockout female mice	Impaired PI3K/NF‐κB pathway	Reduced steroidogenesis‐associated genes star, Hsd3β2, cyp19a1 in GCs, Lhcgr, star, Hsd3β2, cyp17a1 in TCs	[77]
	*Bmal1‐*knockdown GCs	Inactivated PI3K/AKT/mTOR pathway	Reduced synthesis of progesterone and estradiol	[78]
	*Akt1* ^−/−^ mice	Reduced Rev‐ERBα in the heart and liver, increased PER2, Rev‐ERBα, Dbp expression loss of CLOCK expression rhythmicity in aorta	–	[81]
	Hyperosmotic pulse treatment–induced osmotic stress	Increased AKT phosphorylation at S473	Increased Ser‐845 phosphorylation of CLOCK leading to blocked the clock resetting	[82]
	*Clock* S845A knocking mice and HEK293T and NIH3T3 cells	AKT phosphorylates CLOCK at S845	Inhibited CLOCK translocation into the nucleus	[83]
	*Akt2* ^−/−^ mouse line	AKT induces BMAL1 phosphorylation at S42	–	[84]
	Pregnant Wistar rats with sleep deprivation	Decreased AKT phosphorylation, downregulated BMAL1, CLOCK and PER2	Reduced estradiol and progesterone secretion	[111]
	*Bmal1* knockdown TM3 Leydig cell	Reduced the expression of phosphorylated p85 and AKT	Promoted apoptosis and reduced testosterone secretion	[112]
	Per2 gene mutant mice	Inhibited AKT activity	Induced hyperinsulinemia and hypoglycemia and compromised insulin‐mediated endothelial nitric oxide release	[113]
	Valvular interstitial cells isolated from normal and calcified human aortic valves	Knockdown of *Bmal1* led to decreased levels of phospho‐AKT	Reduced valvular cells' osteogenic differentiation	[114]
	Normal mice and Mice with a CD8 T cell‐specific *Bmal*1 deletion	Induced AKT and mTOR activity	Vaccination done during the middle of the day leads to higher T cell activation	[115]
	C3H10 and C2C12 cells with silenced Cry1	Reduced PI3K/AKT activity	Inhibited osteoblast differentiation	[116]
Cancer‐pathologic condition	Lung cancer and glioma cells	BMAL1 blocked PI3K‐AKT‐MMP‐2 pathway	Suppressed cancer cell invasion	[117]
	Colon cancer cells	BMAL1 induced PI3K/AKT pathway activity	Induced epithelial‐mesenchymal transition, invasion and migration	[118]
	Colorectal carcinoma cells	CLOCK promoted AKT phosphorylation and inhibited Bcl2 expression	Inhibited apoptosis of tumour cells	[87]
	Bladder cancer cells	Overexpression of REV‐ERB reduced AKT activity	Suppressed the tumorigenicity of bladder cancer cells	[119]
	Glioblastoma cells	REV‐REBβ induced AXL‐mediated PI3K/AKT activation	Induced tumour cells' proliferation and motility	[91]
	Osteosarcoma cells	Overexpression of PER2 inhibited AKT phosphorylation	Induced Apoptosis of tumour cells	[88]
	Osteosarcoma cells	Overexpression of RNF38 induced Cry‐mediated AKT activation	Induced the proliferation of osteosarcoma cells, the number of colonies	[120]
	Osteosarcoma cells	Cry1 induced AKT activity and inhibited p53	Promotes proliferation and migration of tumour cells	[90]
	Lung adenocarcinoma cells	PER2 knockdown increased AKT activity	Participated in drug resistance in tumour cells	[121]
	Ovarian cancer cells	PER2 suppressed PI3K/AKT pathway	Promoted cisplatin sensitivity in tumour cells	[122]
	Ovarian cancer cells	PER2 suppressed the PI3K/AKT pathway	Promoted cisplatin sensitivity in tumour cells	[123]

## 6 THE PI3K/AKT PATHWAY AND CIRCADIAN RHYTHM COMPONENTS: PATHOLOGIC CONDITION AND PHARMACOLOGIC INTERVENTIONS

Accumulating evidence points to a significant association between the PI3K/AKT signalling pathway and circadian rhythm during cancer progression, which introduce it as a suitable option for pharmacologic interventions. As described previously, there are numerous studies indicating alterations in PI3K/AKT pathway or core clock components, which together results in cancer initiation and progression. However, the interplay between these two pathways in different cancers is mostly investigated in the case of tumour cells' proliferation and invasion. In a study by Chen et al. it has been reported that BMAL1 overexpression in tongue squamous cell carcinoma enhances cell death, decreases cell proliferation, and hinders the migratory ability of these cells. Observed properties from BMAL1 was linked to its ability in autophagy induction as high magnitudes of autophagosomes detected in tumour cells. Evaluation of the expression levels of AKT, p‐AKT, mTOR, and p‐mTOR clearly showed that BMAL1 induced autophagy in tongue squamous carcinoma cells is mediated by activation of AKT/mTOR pathway, which consequently leads to modulation of BAX/Bcl2 and matrixmetalloproteinase‐9 (MMP‐9) as apoptosis‐ and metastasis‐related factors, respectively.^86^ Treatment of U87MG glioblastoma cells with BMAL1 small interfering RNA (siRNA) has been shown to promote the proliferation of tumour cells. Indeed, using a wound healing assay, it was observed that BMAL1 overexpression led to inhibit glioblastoma cells' invasion and migration, which were linked to reduced MMP‐9 expression via AKT inhibition.^63^ Regarding CLOCK transcription factor, it has been reported that its upregulation results in SW480 colorectal cancer cell proliferation, which has been linked to AKT‐mediated Bcl‐2 suppression.^87^

A couple of studies have investigated the effects of other circadian clock components on proliferation, apoptosis, and metastasis of different types of cancer. Overexpression and downregulation of PER2 in osteosarcoma cells have been shown to reduce proliferation and induce apoptosis in tumour cells, respectively, as well as affected tumour cell migration. Further analysis indicated that PER2 downregulation was accompanied with a significant decrement in AKT phosphorylation at S473, which is an activation site and contributes to increased Bcl‐2 and cleaved caspase‐3 levels.^88^ In another study, it was shown that the expression of PER2, PTEN, and p53 were significantly reduced in oral squamous cell carcinoma tissues, while the protein levels of PIK3CA exhibited a marked increase. Moreover, PER2 expression was positively correlated with PTEN, and caspase‐8 levels, and negatively correlated with PIK3CA and p53 levels, indicating an alteration that leads to increased tumour‐invasion.^89^ Knockdown of Cry1, the other clock component, has been reported to induce the proliferation of osteosarcoma cells. This observation was linked to its effect on AKT activity, as it was indicated that Cry1 knockdown activates the AKT/p53/p21 pathway and increases the levels of mouse double minute 2 (Mdm‐2), which is responsible for sensitivity to chemotherapy.^90^ It has been reported that REV‐REBβ, which is overexpressed in human glioblastoma, inhibits migration, invasion, and proliferation of glioblastoma cells. The results of this study revealed that REV‐REBβ knockdown leads to a significant decrement in the levels of the receptor‐tyrosine kinase AXL. This observation may present a new insight into how core clock components regulate PI3K/AKT activity in tumour cells. Interestingly, further analysis indicated that REV‐REBβ‐mediated AXL upregulation leads to PI3K/AKT pathway activity leading to enhance proliferation and motility of glioblastoma cells.^91^

## 7 CONCLUSION

Increasing evidence links alterations in the activity of the PI3K/AKT pathway and circadian rhythm to cancer development. It is clearly understood that the PI3K/AKT signalling pathway regulates the activity of components involved in circadian rhythms via regulation of their expression, degradation, and translocation into the nucleus. In turn, different core clock regulators regulate the activity of the PI3K/AKT pathway. However, there is no information indicating which one may be altered first to lead a disturbance in the activity of the other one. As mutations in different isoforms of PI3K and AKT have been detected in various cancers, it may be concluded that circadian rhythm alteration in cancer may be presented as a result of PI3K/AKT dysregulation. However, modern life style with disturbed day/night cycles may contribute to cancer initiation and progression via its effect on intracellular factors, especially the PI3K/AKT pathway. This supports the hypothesis while our genome and resulting proteomes can adapt to changing life conditions during long periods, such altered circadian rhythms can trigger pathologies such as diabetes and cancer, and this issue warrants further investigation in future studies.

## AUTHOR CONTRIBUTIONS

Mohammad Rafi Khezri: Writing manuscript and visualization; Somayeh Mohammadipanah: Data collection. Hsiang‐Yin Hsueh: Editing. Javad Khalili Fard: Revision. Morteza Ghasemnejad‐Berenji: Supervision, Final review and revision of the manuscript.

## CONFLICT OF INTEREST STATEMENT

The Authors declare no competing financial or non‐financial interests.
